# Leafhopper males compensate for unclear directional cues in vibration-mediated mate localization

**DOI:** 10.1038/s41598-023-35057-z

**Published:** 2023-06-01

**Authors:** Jernej Polajnar, Anka Kuhelj, Rok Janža, Nada Žnidaršič, Tatjana Simčič, Meta Virant-Doberlet

**Affiliations:** 1 Department of Organisms and Ecosystems Research, National Institute of Biology, Večna pot 111, Ljubljana, Slovenia; 2 Department of Biology, Biotechnical Faculty, University of Ljubljana, Jamnikarjeva 101, Ljubljana, Slovenia

**Keywords:** Animal behaviour, Entomology

## Abstract

Ambient noise and transmission properties of the substrate pose challenges in vibrational signal-mediated mating behavior of arthropods, because vibrational signal production is energetically demanding. We explored implications of these challenges in the leafhopper *Aphrodes makarovi* (Insecta: Hemiptera: Cicadellidae) by exposing males to various kinds of vibrational noise on a natural substrate and challenging them to find the source of the female playback. Contrary to expectations, males exposed to noise were at least as efficient as control males on account of similar searching success with less signaling effort, while playing back male–female duets allowed the males to switch to satellite behavior and locate the target without signaling, as expected. We found altered mitochondrial structure in males with high signaling effort that likely indicate early damaging processes at the cellular level in tymbal muscle, but no relation between biochemical markers of oxidative stress and signaling effort. Analysis of signal transmission revealed ambiguous amplitude gradients, which might explain relatively low searching success, but it also indicates the existence of behavioral adaptations to complex vibrational environments. We conclude that the observed searching tactic, emphasizing speed rather than thorough evaluation of directional cues, may compensate for unclear stimuli when the target is near.

## Introduction

In the broadest sense, the term »ambient noise« comprises any perturbation in the medium that diminishes the receiver's ability to detect a signal or discriminate between signals^1^. Along with inherent properties of the medium, such as the rate of attenuation, noise is one of the most important environmental factors determining signal active space, a key concept in studying animal communication^1–3^. In recent years, vibrational communication is emerging as a useful system for exploring effects of such environmental constraints on communication. While conceptually and evolutionarily related to the far better-known acoustic communication^4^, vibrational communication nevertheless poses a unique set of benefits and constraints to any animal utilizing it^5^. Here, many basic questions still remain open, among those the influence of noise which few studies have addressed specifically.

The use of vibrations for communication is the most prevalent and best researched in plant-dwelling insects^6^. Vibrational active space is largely limited to continuous substrate, in this case the plant the animal is standing on, as well as to neighboring plants in physical contact^3^. Plants impose a low-pass filter, rapidly attenuating any frequency components above the Kilohertz range^7^. At the same time, the lowest-frequency range is occupied by pervasive ambient noise, thus channeling vibrational communication of plant dwellers within the frequency range between few tens of Hz and few KHz^6,8–10^. Lastly, the finite physical structure of stalks and leaves gives rise to resonance, so attenuation of individual frequency components is non-monotonous and moving closer to the source may not reliably increase signal-to-noise ratio^11^. Within these constraints operates a potentially diverse local community of signalers, intended receivers and eavesdroppers inhabiting the landscape^10,12^.

Considering the network aspect of vibrational community, three general categories of noise in vibrational environment can be distinguished: abiotic, biotic and anthropogenic, each with its own temporal and spectral properties^9,10^. Biotic noise, produced by other signalers using the same medium at the same time within detection range of the intended receiver, is presumed to be one of the most important factors limiting vibrational communication in the wild because the narrow available frequency band limits options for avoiding interference in the spectral domain. Field data are scarce due to technical challenges, but it appears that, for example, small Auchenorrhyncha represent a major source of vibrational emissions in temperate hay meadows^9,10,13^. Within Auchenorrhyncha, leafhoppers from the genus *Aphrodes* Curtis, 1833 (Hemiptera: Cicadellidae) are used as model organisms in studies of vibrational interactions^12,14,15^ as well as energetics^16^ and other topics in biotremology, not least because mating behavior in leafhoppers is mediated exclusively by vibrational signals^17^, simplifying interactions. *Aphrodes* species are an important part of leafhopper communities in grasslands of the Palearctic^18^, and field recordings in a temperate hay meadow in southern Central Europe show that their signals are likewise an important constituent of the vibrational environment^10^. A complex of cryptic species is recognized under the name *Aphrodes bicincta* (Schrank, 1776) s.l., including *A. makarovi* Zachvatkin, 1948 and the *A. bicincta* ‘Dragonja’ type which has not yet formally described as a new species^14,19^. These leafhoppers are closely related to *A. bicincta*, but strong female preferences for distinct conspecific male advertisement calls result in reproductive isolation^14,19,20^. While *Aphrodes* species differ in their ecological requirements, they are often found syntopic at the same site^20^, and have a univoltine life cycle with a relatively short reproductive period^21^, thus vibrational interactions are likely.

The duet of *A. makarovi* Zachvatkin, 1948 consists of a multi-component male signal followed by a monotonous female reply that partly overlaps the final section of the male signal^14,22^. Male signals are composed of four distinct sections, Me0–Me3, of which Me3 is necessary to trigger the female reply, while the presence of others can vary in male song^14^. Males use the typical ‘fly/jump/walk-call’ strategy^22,23^, signaling spontaneously from different positions and searching on foot after receiving the female vibrational reply, using directional cues in replies to locate her on the host plant. A vibrational duet is thus a prerequisite to mating. Vibrations are generated by a tymbal or tymbal-like mechanism on the first abdominal segment to which different muscles are attached^24,25^. The main dorsoventral muscle (Ia dvm_1_) is presumed to be homologous to the tymbal muscle of cicadas (Hemiptera, Cicadidae), which belong to the same infraorder Cicadomorpha^24,25^. In cicadas, tymbal muscles are recognized as fast muscles rich with sarcoplasmic reticulum and mitochondria, attesting to high energy demand^26–28^.

*A. makarovi* males exhibit different patterns of activity throughout their lifetime, and various alternative mating tactics have evolved, such as emission of masking signals and satellite behavior. Crucially, mating success is determined by locating the female first, regardless of signaling effort in case of male-male conflict^15^. Basic mechanisms of vibration-mediated mate recognition and localization in duetting insects have been unraveled in recent years, either by observing duetting couples or simulating one of the partners with playback and carefully recording behavioral parameters^14,29–33^. However, the impact of broader vibrational environment with competitors and other external sources of noise has been largely neglected so far^12^, with some exceptions^15,34^.

Introducing various kinds of vibrational noise with behaviorally relevant amplitude, frequency and temporal characteristics to a well-characterized system and observing the response may serve to evaluate practical significance of emergent phenomena such as satellite male tactics, thus bridging insights from laboratory experiments with isolated animals and processes on a community scale. Impact of noise on behavior can be measured directly, whereas its long-term effects can be inferred from physiological markers associated with oxidative stress^35–38^. Playback experiments have demonstrated that in *A. makarovi* males high signaling effort in the first 3 weeks of adult life has negative effect on longevity, where the authors suggested that high mortality is likely to result from energetically demanding vibrational signaling and associated indirect costs of sexual signalling^16^. Reactive oxygen species (ROS) produced in mitochondria during energy production are a proposed mechanistic explanation for the association between signaling effort, oxidative stress and aging in insects; although, the relationship is not necessarily direct^36,39^. Oxidative damage to proteins is nevertheless considered a key indicator of oxidative stress, typically expressed as protein carbonyl content^36,38^. In addition to proteins, membrane phospholipids are also exposed to oxidants that cause lipid peroxidation, which is typically measured by the amount of malondialdehyde (MDA) accumulated in the brain^37,40,41^. Oxidative stress can accelerate aging and cause mortality in a number of organisms^35–38^, so linking increased signaling effort in response to vibrational noise with indicators of oxidative stress could serve to quantify the role of vibrational noise in natural selection. Analysis of cell ultrastructure may provide additional clues, particularly the evaluation of mitochondrial architecture, as mitochondria are the key generators of cell energy and crucially involved in the mechanisms of cell function and survival control. Mitochondrial structure and function dynamically reflect the cell physiology and changing energy demands^42,43^ as well as pathological states and external stresses^44–46^. It is extensively evidenced that mitochondrial ultrastructure and function are intimately linked. Mitochondrial architecture adapts dynamically to bioenergetic state and it is remodeled also in concert with non-bioenergetic functions of mitochondria, such as cell signaling through reactive oxygen species production^47^.

We hypothesized that different types of external vibrations would differently affect mate localization by *A. makarovi* males, depending both on their type and the proportion of the female reply masked. We predicted that masking vibrations would diminish the efficiency of localization by forcing the males to invest more effort in signaling and searching, but conspecific masking signals would be recognized as such by the male, which would be reflected in shorter response latency compared with heterospecific signals and anthropogenic noise. On the other hand, efficiency would be improved when exposed to duets, enabling the males to switch to eavesdropping and exploiting male–female vibrational interaction for silently approaching the source of female reply (i.e. satellite behavior)^15^. Behavioral parameters related to effort were in turn predicted to be positively correlated with biochemical markers of oxidative stress and ultrastructural alterations in tymbal muscle, thus linking noise in the communications channel with ageing. As an alternative hypothesis, males could exploit uneven dispersion of vibrations and gaps in masking to reduce the effect of masking, in which case the relation between masking, efficiency, effort and markers of oxidative stress would not be apparent.

## Results

### Behavioral experiments

**Figure 1:**
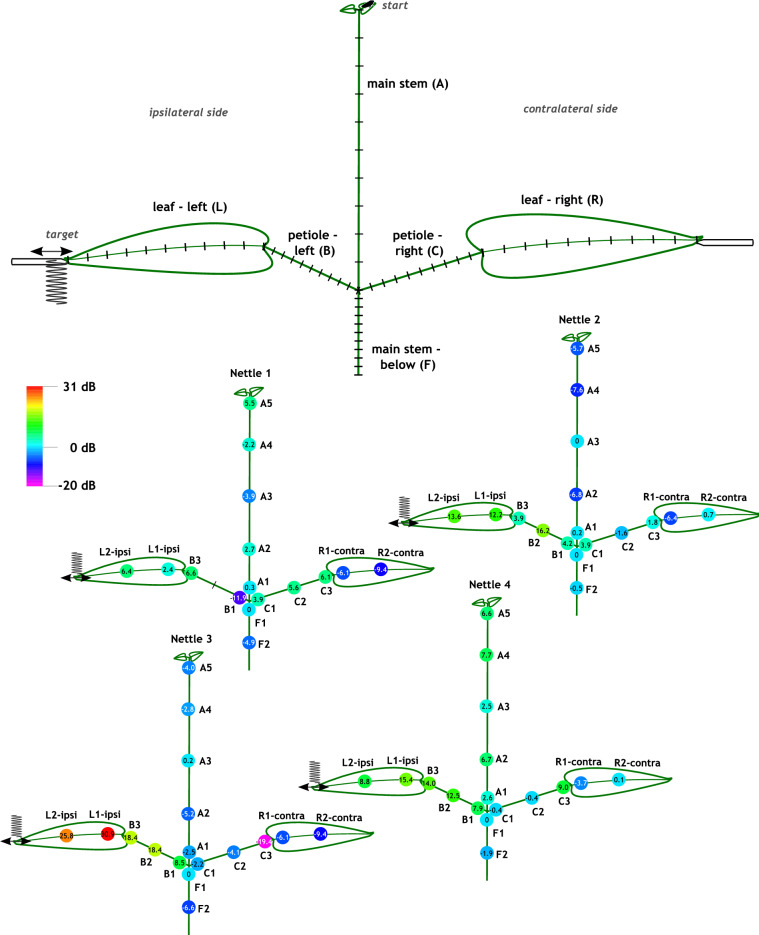
Above: diagram of the experimental arena depicting the starting position of the male (“start”), attachment of both shakers, and division of each section to 10 subsections for analysis of movement. The leaf with a shaker playing the female reply was designated ipsilateral (target), while the other one was contralateral; the sides were randomized for each trial. Below: transmission of vibrations along the four measured plants, played from the left shaker (ipsilateral; plane of movement denoted by grey arrow, tip of the shaker shown). The contralateral leaf had an inactive shaker attached. Average amplitude for each section of the arena is shown, calculated from measurements of equivalent locations on four nettle cuttings. All values refer to the semi-major axis of the ellipse described by movement of stalks and stem at 430 Hz – dominant frequency of female song playback – expressed in dB relative to the value at F1. F1 was taken as a reference point because it was closest to the location where a male had to make a directional choice and was also used for recording vibrations in behavioral trials. Plant parts not completely to scale, measurements are given in Supplementary Data S1.

Males were tested in six experimental situations with a minishaker on one leaf playing female reply to each male’s signal and one on the other leaf playing the noise treatment (Table 1). The males were challenged to find the source of the female playback at the tip of the target minishaker. Results are expressed in terms of overall efficiency, which is used to compare treatments. We defined males’ overall efficiency as a ratio between a measure of signaling effort and a measure of path efficiency, which evaluated searching movements on the host plant which served as the arena (Fig. 1). Maximum overall efficiency (eff = 1) would mean walking straight and all the way to the target location – a minishaker playing the female reply – without signaling. We did not find significant effect of operator on overall efficiency or any of the parameters this measure was composed of (ANOVA, p > 0.05), so results are pooled. Summary descriptions of differences between treatments (Tukey, or ANOVA for parameters where treatment was not found to have an effect) are reported here for brevity, whereas tables for post-hoc tests are given in Supplementary Data S3.

**Table 1:** Design of playback experiments.

Treatment	Short description	Ipsilateral playback	Contralateral playback	N
“Control + ”	Just female reply	Female reply triggered manually during the last phase of the male’s Me3 signal section	–	13
“Rival”	Rivalry	Female reply as in “control + ”	Male masking signal triggered automatically 4.1 s before the end of the male signal and continuing 1.4 s after its end	13
“Duet”	Duet	Female reply triggered automatically 1.5 s before the end of the male signal and continuing 8.5 s after its end	Male advertisement signal looped with 12.1 s pause between consecutive signals	14
“Bionoise”	Heterospecific signals	Female reply as in “control + ”	*A. bicincta* 'Dragonja' male advertisement signal; looped with 10 s pause between consecutive signals	13
“Anthronoise”	Anthropogenic noise	Female reply as in “control + ”	Recording of lawnmower vibrations picked up by plants in the field; looped continuously	15
“Control- “	No playback	–	–	15

We observed little or no movement and signaling in males in the negative control group (“control–”, no playback from either minishaker): median movement distance in 72 pooled “control- “ trials with 15 males was only 30 mm, only two “control–” trials (2.8%) ended with the male reaching the silent target shaker, and median number of signals was 1. Both movement distance and signaling duration were also significantly lower in “control–” than in all other treatments (Wilcoxon rank sum test with continuity correction, p < 0.0001). Therefore, we did not compare overall efficiency or other parameters of “control-”with other treatment groups.

**Figure 2:**
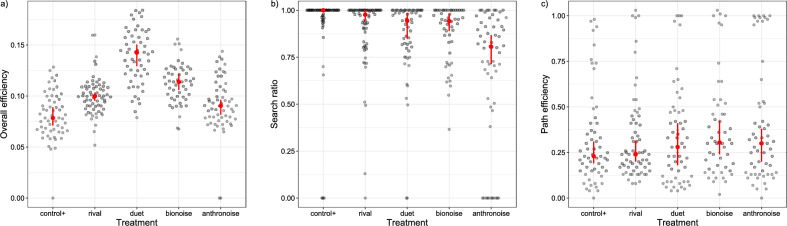
Dot plots showing (**a**) overall efficiency; (**b**) search ratio and (**c**) path efficiency of males exposed to different treatments. Red star is median together with 95% confidence interval around median (red line). Labels denote different treatments: (control +) female reply only, (rival) female reply + masking signal, (duet) male signal + female reply loop, (bionoise) *A. bicincta ‘Dragonja’* noise, (anthronoise) anthropogenic noise.

**Figure 3:**
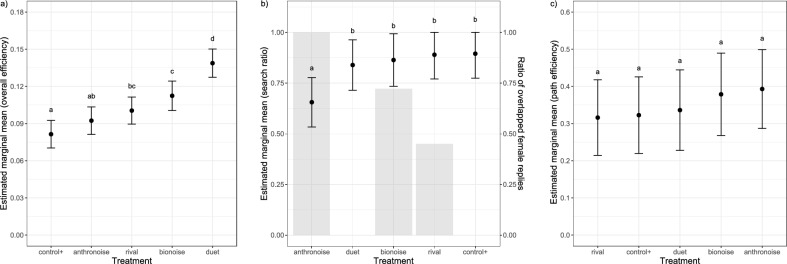
Estimated marginal means together with 95% confidence intervals for (**a**) overall efficiency; (**b**) search ratio; (**c**) path efficiency. For (**b**) search ratio, the mean ratio of female reply cover by noise is also shown with grey bars. Distributions not sharing any letter are significantly different by the Tukey method at 5% level of significance.

Among treatments with playback, the males in the positive control (“control + ”, female reply only) exhibited lowest overall efficiency, significantly lower than both biotic noise treatments (“rival” and “bionoise”), whereas the males in the “duet” treatment exhibited the highest overall efficiency. On the other hand, overall efficiency of males exposed to anthropogenic noise (“anthronoise”) was also low, not significantly different from “control + ” (Tukey, p < 0.05; Figs. 2a, 3a). Overall efficiency thus did not correspond with the percent of the female reply masked: 0% in “control + ”, 45% in “rival”, 72.2 ± 16.8% in “bionoise” and 100% in “anthronoise” treatments. Searching success (i.e. percentage of males which found the target shaker) was highly variable and treatment had no effect (ANOVA, p = 0.432); males found the target shaker in 123 out of 346 pooled trials (35.5%).

To disentangle causes for such a result, we looked at basic behavioral parameters of moving males.

**Figure 4:**
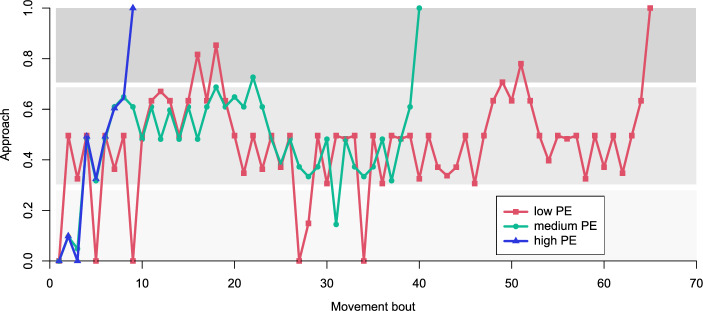
Path analysis of three exemplar males with different path efficiencies (PE): high (0.65), medium (0.20) and low (0.09), expressed as percentage of approach to the target shaker. Background shading approximately denotes sections of the experimental arena (bright: tip of the stem and contralateral leaf, medium: stalks and sections of stem close to the branching point, dark: ipsilateral leaf).

Path analysis of representative trials revealed that the area around the crossing accounted for the majority of wrong directional decisions in cases of low path efficiency (Fig. 4). However, treatment was not found to have an effect on path efficiency (ANOVA, p = 0.4235; Figs. 2c, 3c), nor on moving (ANOVA, p = 0.094) and searching latency (ANOVA, p = 0.088) in treatments with playback. Similarly, exposure to noise did not affect searching success: approach to target shaker did not differ from “control + ” (Tukey, p > 0.05), nor did time needed to reach the location closest to the target (Tukey, p > 0.05). The group “anthronoise” had significantly lower search/walking ratio than all other treatments exposed to playback (Tukey, p < 0.05; Figs. 2b, 3b). A trend of decreasing search/walking ratio with increasing noise overlap was discernible (Fig. 3b). Plant ID significantly influenced moving parameters.

Total signaling duration in “duet” and “bionoise” treatments was significantly lower than “control + ” (Tukey, p < 0.05), as was duty cycle (Tukey, p < 0.05). Treatment had no effect on average male signal duration (ANOVA, p = 0.901). Furthermore, treatment did not affect proportion of Me3 signal section duration in signal duration (ANOVA, p = 0.798). Some males responded with emission of masking signals, but the proportion of masking signal duration in total signal duration was only significantly higher in the group “duet” (Tukey, p < 0.05). The same group also had significantly higher proportion of interrupted calls than all other groups, on the account of stimulation with the pause shorter than the normal signal duration (Tukey, p < 0.05). Male ID significantly influenced signaling parameters.

Males in the group “bionoise” were exposed to highly varying noise levels which, while occurring in a simple loop, were assumed to be unpredictable by the tested male. The males responded to this sequence by tending to start their signals within 10-s silent periods between *A. bicincta* 'Dragonja' signals which comprised the “bionoise” playback – in pooled data, a quarter of signals (25.5 ± 24.9%, N = 66, n = 750) started during active noise playback, whereas approximately 60% would be expected according to the noise's duty cycle if onset was random. On average, 72.3 ± 16.4% (N = 66, n = 750) of signal duration was covered by noise.

Characteristics of the “bionoise” playback (Table 1) meant that the reply playback was often overlapped by noise to some extent because the male signal tended to start in the silent period. Usually, the reply started when *A. bicincta* ‘Dragonja’ playback was still ongoing and continued unmasked for several seconds after the *A. bicincta* ‘Dragonja’ signal ended, but could also be entirely masked or entirely unmasked. This extent was measured, averaged for the whole trial and correlated with behavioral parameters (Pearson’s product-moment correlation). Exploratory analysis using pooled signal measurements from all males in the group “bionoise” showed positive correlation between overall efficiency and the average proportion of masked reply duration within a trial (r_(48)_ = 0.34, p = 0.017). Seemingly, this result was on account of negative correlation between average proportion of masked reply duration and total signal duration in a trial (r_(60)_ = − 0.29, p = 0.024), as well as between average proportion of masked reply duration and duty cycle (r_(60)_ = − 0.44, p < 0.001). Furthermore, proportion of masked reply duration was positively correlated with Me1/signal ratio (r_(47)_ = 0.30, p = 0.036) and Me2/signal ratio (r_(47)_ = 0.50, p < 0.001), and negatively correlated with Me3/signal ratio (r_(47)_ = − 0.33, p = 0.021) implying that Me1 and Me2 was prolonged on account of Me3. However, repeated measures correlation (calculated with R package rmcorr 0.4.3^48^) revealed no within-individual correlation between average proportion of masked reply duration and total signal duration (r_(35)_ = 0.05, 95% CI [− 0.28, 0.38], p = 0.73).

### Signal transmission

Played-back female signals did not reliably follow a trend of increasing amplitude with decreasing distance to the vibrational exciter (Fig. 1, Supplementary Data S1).

As a general observation, the highest amplitudes were measured on the ipsilateral leaf and stalk, while the bottom of the stem (below the junction) always vibrated with lower amplitude than the reference point. No other rule could be discerned; a male moving across the surface of tested nettle cuttings like in behavioral experiments (Fig. 4) would encounter fluctuating amplitude of the female reply regardless of direction and distance to the source.

### Tymbal muscle ultrastructure

**Figure 5:**
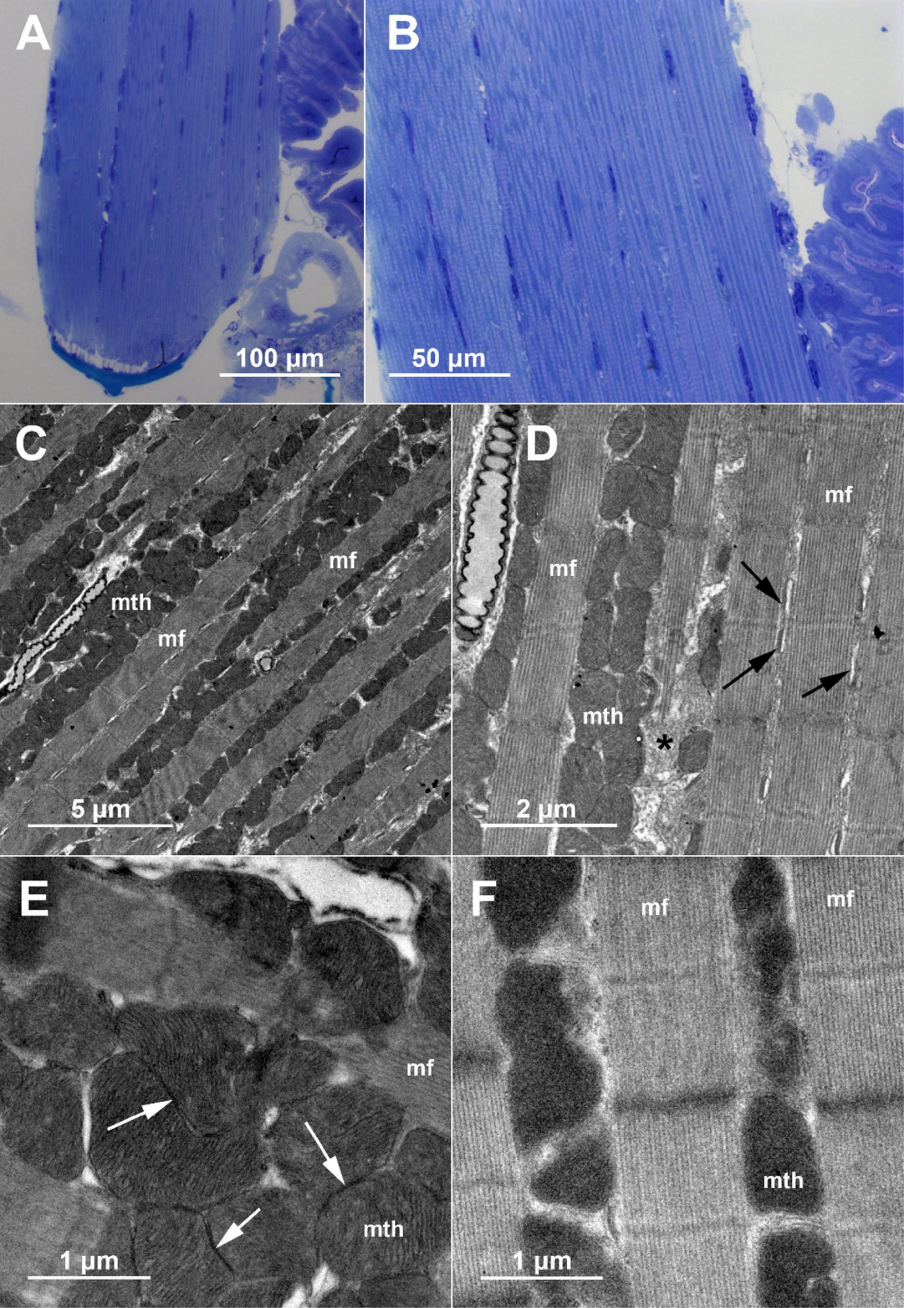
Structure of the tymbal muscle (Ia dvm_1_) in control and treated males. (**A**,**B**) general structure as viewed on semi-thin section by light microscope. (**C**–**E**) ultrastructure of the tymbal muscle in a control male with low signaling effort, exhibiting closely apposed mitochondria with densely packed cristae and intermitochondrial junctions. (**F**) ultrastructure of the tymbal muscle in a treated male with high signaling effort, where mitochondrial cristae were hardly discernable or not evident and intermitochondrial junctions were less abundant or not apparent. Labels: *mf* myofibrils, *mth* mitochondria, *tr* tracheola, asterisk – sarcoplasmic reticulum (SER), black arrows – complexes of T-tubules and SER, white – arrows intermitochondrial junctions.

The analyzed tymbal muscles of *A. makarovi* were characterized by abundant mitochondria, extensive sarcoplasmic reticulum (SER) and numerous tracheoles (Fig. 5A,B). Complexes of T-tubules and SER were prominent and located on both sides of the M line. Large mitochondria were aligned longitudinally in rows between myofibrils (Fig. 5D). In general, mitochondria harbored numerous densely packed cristae, electron dense matrix and were closely apposed to each other. Adjacent mitochondria exhibited enhanced electron density at closely apposed surfaces characteristic for intermitochondrial junctions (IMJ) (Fig. 5E) described in recent studies on mammals.

In both untreated (»control–«) males, cristae in mitochondria were clearly discernible (Fig. 5C–E), different from treated males, where cristae were in general hardly discernible or not evident (Fig. 5F). IMJs were frequent in untreated males, but less numerous or not evident in males exposed to noise.

### Analysis of oxidative damage

Carbonyl concentration in tymbal muscle tissue was 4.2–18.0 nmole carbonyl/mg protein (median: 6.6, N = 93). Control (young) and experimental (older) males did not differ in carbonyl concentration (median_Contr_: 6.0, n = 14, median_Exp_: 6.8, n = 79; Mann–Whitney test: Z = 0.99, p = 0.32), neither did the concentration differ between experimental treatments (ANOVA: F_(5,71)_ = 0.69, p = 0.63). Carbonyl concentration did not correlate with signaling effort (t = − 0.93, df = 76, p = 0.35).

Malondialdehyde concentration in brain tissue was 13.7–193.9 nmole MDA/g WW (median: 71.3, N = 93). Control (young) and experimental (older) males did not differ in MDA concentration (median_Contr_: 74.8, n = 14, median_Exp_: 70.8, n = 79; Mann–Whitney test: Z = 0.93, p = 0.35), neither did the concentration differ between experimental treatments (ANOVA: F_(5,71)_ = 1.56, p = 0.18). MDA concentration did not correlate with signaling effort (t = − 0.95, df = 76, p = 0.35).

## Discussion

In the present study, we studied male signaling and mate localization in the leafhopper *A. makarovi* under simulated noisy conditions on a natural substrate, predicting that interference in the vibrational channel will diminish efficiency by hampering mate recognition and localization in *A. makarovi* males, requiring more effort to locate the female. This idea expands the work on *Scaphoideus titanus* Ball where rival masking noise has been shown to hinder sexual communication and prevent mating in laboratory and semi-natural settings^49,50^, but also other contemporary work on the impact of vibrational noise on sexual communication of Hemiptera^29,34,51,52^. Contrary to the original hypothesis, males exposed to biotic noise showed even higher overall efficiency than control males which only received female replies, whereas males exposed to continuous anthropogenic noise were just as efficient as control males. The former was on the account of similar path efficiency with less signaling (lower duty cycle), while the latter group also exhibited significantly more non-searching movements. Other groups exposed to noise, either conspecific masking signals or interspecific signals, showed a slight trend of increasing proportion of non-searching movements with increasing masking. This is consistent with the alternative hypothesis, but high variability of transmission precludes firm conclusions. On the other hand, males that were able to exploit a duet exhibited highest overall efficiency on account of being able to locate the source of female reply without having to signal themselves, as predicted. No effect of treatment or signaling effort on biochemical markers of oxidative stress was found, but highly active males showed signs of oxidative damage to mitochondrial structure of tymbal muscle cells.

Localization of the signal source is a key issue in long-range mating behavior. Direction of wave propagation seems to be the most important cue for localization in treehoppers from the genus *Umbonia* (Hemiptera: Membracidae)^32^; treehoppers may be able to extract this cue directly on account of directional mechanical response of their body^53^, so directional decisions are fairly accurate, with accuracy improving closer to the vibrational source^32^. Increased accuracy closer to the source was also reported by for *Tylopelta* treehoppers^52^. This sampling strategy resembles stink bugs (Hemiptera: Pentatomidae) which are large enough to be able to detect time delay between the arrival of vibrational wave to receptors in legs^54^, but it has not been observed in *Scaphoideus* leafhoppers which were equally likely to make a correct or wrong directional decision at a branching point, then reversed the direction in case of a wrong turn^30^. In the present study, more active males made dozens of turns and direction reversals, walking repeatedly across the same sections of the test plant if they failed to locate the source of the female reply early. Therefore, we did not characterize vibrations at each stopping point; instead, transmission of female reply playback at the dominant frequency was measured on a different nettle cutting with similar dimensions as a proxy. The measurements revealed that amplitude of the female reply did not correlate with distance from the source, which could partly explain low searching success, particularly in the positive control group which received only female reply. The vibrational environment was thus less predictable than, for example, in experiments on grapevine cuttings where the monotonous decrease of amplitude away from the source enabled precise characterization of the pre-mating sequence in *Scaphoideus titanus*^30^. The cause of this discrepancy is unclear. Moreover, manual stimulation with prerecorded female reply could not fully emulate the changing sender-receiver dynamic during *A. makarovi* duetting^22,31^. Male response to continuous anthropogenic noise was similar to that observed for abiotic noise^34^; although, neither study examined live duets.

General ultrastructural organization of tymbal muscles in *A. makarovi* exhibited characteristics reported for tymbal muscles in cicada^26–28^. Numerous mitochondria, arranged in rows between the myofibrils, and abundant sarcoplasmic reticulum are the two most conspicuous hallmarks of tymbal muscle cells. Extremely numerous, densely packed cristae, evident in control samples, are in concordance with high energy demand of these cells. It is known that mitochondrial cristae modulations and perturbations correspond to different physiological and pathological conditions^55^. In samples of *A. makarovi* males exhibiting high signaling effort, the alteration of internal mitochondrial structure in tymbal muscle is indicated, as cristae were not clearly discernable, pointing to processes of cristae disintegration. Disintegration and loss of mitochondrial cristae was reported as an aging-related change in *Drosophila melanogaster* and fungi^43,56,57^. As disorganization of crista membrane architecture was linked to a functional decline of mitochondria in aged flies^57^, the observed alteration in *A. makarovi* males with high signaling effort may be associated with indirect costs of sexual signaling with delayed negative effects that affect lifespan^16^.

In tymbal muscle of untreated males, the closely apposed surfaces of adjacent mitochondria frequently exhibited enhanced electron density, which is characteristic for intermitochondrial junctions (IMJs) studied recently in mammals^58–60^. The examination of some invertebrate muscle micrographs also revealed the presence of IMJs^59^, but no comparable reports on invertebrate muscles are available. According to our observations, IMJs are frequent in the tymbal muscles of control males, while a disruption of IMJs is suggested in treated males with high signaling effort. From studies on mammals, it is known that enhanced cellular energy demand in mouse skeletal muscle during voluntary exercise is associated with increase of IMJ prevalence^61^. Interestingly it was demonstrated in mouse skeletal and cardiac muscles that physical separation of malfunctioning mitochondria occurs by detachement of IMJs and mitochondria reshaping, which is interpreted as a protection mechanism against dysfunction spread throughout the mitochondrial reticulum in the cell^62^. We therefore surmise that by evoking high signaling effort which is energetically demanding^16^, we triggered perturbations in mitochondrial structure that likely indicate early damaging processes at the cellular level, but we were unable to confirm higher ROS concentration in the muscles of the most active males. Lack of differences in oxidative stress markers between treatments could be attributed to insufficient sensitivity of the method, since negative control – young untreated males – did not differ either. Alternatively, it could be a consequence of spontaneous signaling activity^16,22^ which we could not control or measure outside of trials.

Results of the trials reported here reveal alternative behavioral mechanisms of leafhoppers that enable mate localization in environments where locational information is obscured by the medium or ambient noise without significantly higher effort. The chosen arena – a nettle cutting – represents a realistic natural setting which leads to thinking that we should be especially careful about generalizing insights from laboratory experiments done in artificial arenas in biotremology. Our observations indicate that males of *A. makarovi* are able to compensate for unclear stimuli by simply running across the substrate and trying as many turns as possible. Such a tactic can work in a relatively small arena such as ours, and may paradoxically lead to even higher overall efficiency than a more thorough approach with testing the amplitude or some other parameter at every stop. Males of the leafhopper *Graminella nigrifrons* Forbes, 1885 also combine vibrational cues with positive phototaxis for locating females perched near the tip of the host plant^23^ which was not yet observed in *Aphrodes*, nor did males in our positive control trials fail to locate the source below their initial position.

We confirmed gap detection on short time scales in *A. makarovi*, as expected from work on other hemipterans^10,13,34^, though its effect on the outcome was diminished due to the nature of our stimulation protocol – looped allospecific song tended to overlap female response whereas live *A. bicincta* 'Dragonja' would likely not initiate a signal during perceived vibrations by *A. makarovi*. The proportion of overlapped reply was correlated with male signal parameters, but not within the same male – meaning that some males’ innate rhythm was better for ensuring low masking of female reply with given playback whereas males did not alter their signal composition in response to noise. The motor pattern responsible for generating signals thus appears to vary in response to duetting dynamic only^31^, and is less plastic than for example the acoustic song of the tree cricket *Oecanthus pellucens* which was shown to alter echeme number in response to fluctuating traffic noise^63^, or the field cricked *Gryllus bimaculatus* which decreases the echeme rate in response to constant traffic noise^64^. Rival masking may also function indirectly, e. g. by decreasing female response^52^, but this was not studied. Conspecific masking signals might also increase the focal male’s motivation because they signify that a competitor is near, but the analyzed behavioral parameters such as latency did not support that possibility.

The caveat of repeated presentation of the same stimulus exemplar is limited generality when conclusions are applied to natural situations^65,66^; although, in the present study spectral variability has been introduced by the experimental plants (see Supplementary Data S1). The present study represents only the first step toward understanding the complex natural situations in which vibrational communication takes place and further studies are needed to extend our knowledge on how animals cope with varying environmental factors during vibration-mediated mating behavior. Males in the population can exhibit different reproductive strategies, with some males signaling intensively at early age and dying early, while others conserve effort to achieve longer lifespan^16^. Similar differences in tendency to exhibit alternative mating tactics are also likely, so further work should explore the effect of such innate differences between male personalities, to reduce variability and possibly reveal additional behavioral mechanisms. Finally, additional measurements of vibrational transmission directly coupled with path analysis would help to shed further light on the searching strategy in small leafhoppers.

## Materials & methods

### Collection and rearing

The animals were collected in early summer 2019 as late-stage nymphs in patches of nettles (*Urtica dioica* L.) at various localities on the southwest edge of Ljubljana Marsh in Central Slovenia. The species was provisionally determined using morphological and ecological characters^20^, then confirmed later in the first behavioral trial series by the emitted vibrational signals. Males that did not react to stimulation by starting to sing during the initial trial (see the section Behavioral trials) were excluded from the experiment and replaced.

The animals were kept in the laboratory of the National Institute of Biology, Ljubljana, at room temperature and 16:8 day/night cycle on alfalfa (*Medicago sativa* L.) cuttings that were replaced twice per week. After final eclosion, the adults were sexed and each male isolated in a separate container with alfalfa.

### Behavioral experiments

We performed a set of playback experiments with *A. makarovi* males on a natural substrate and observed various behavioral parameters that indicate recognition and localization efficiency.

#### Arena

A freshly cut nettle shoot (*Urtica dioica* L.) was picked in the morning before the trials at a forest edge near the laboratory, and used for a whole group of males over the course of 1 or 2 days (see the section “Trials” below), then measured before discarding. The cutting was trimmed so that the stem was nearly smooth and only two opposite leaves remained (Fig. 1). All the cuttings were selected to be of comparable size, with approximately 10 cm long stem above the stem-stalk crossing and 8 cm long leaves on 3 cm long stalks (Supplementary Data S1). On top, the youngest leaves (5–10 mm long) were left for easier placement of the male.

Just below the tip of each leaf, a horizontally positioned minishaker (Type 4810 10N, Brüel & Kjær, Denmark) was attached using office putty, and used for playback (Fig. 1).

Transmission of vibrations across the experimental arena was analyzed on four nettle shoots comparable in dimensions to those used in behavioral trials (Supplementary Data S1). A female signal was played and its amplitude measured successively at different points on the cutting, using a laser vibrometer (PDV 100, Polytec GmbH, Waldbronn). On stalks and stem, the amplitude was measured in two perpendicular planes, both perpendicular to the rod, then major and minor axes of stem motion in 2D at the playback’s dominant frequency (430 Hz) were calculated using the atan2 function^67^. On the leaves, the amplitude was measured in a single plane perpendicular to the surface. Amplitudes at locations along the plant were expressed in dB, relative to the location where vibrations were recorded in behavioral trials (see the section “Trials” below).

#### Trials

Behavioral experiments were conducted in July 2019. Trials started 2 weeks after male eclosion when sexual activity starts^16^. Males from the laboratory population were haphazardly assigned to one of the six treatment groups, labeled individually and kept isolated.

One leaf of the test plant was randomly chosen as ipsilateral (“ipsi”) in each trial. The female reply was played to that leaf, and the other stimulus (if applicable) to the opposite leaf (contralateral, or “contra”), except in negative control where both were silent (Table 1). The male was challenged to locate the target point on the ipsilateral leaf where the minishaker was attached near the tip (Fig. 1).

Playback signal characteristics are given in Table 2.

**Table 2:** Characteristics of playback signals.

Playback signal	Description	Duration [s]	Peak frequency (range) [Hz]	Origin
Female reply	Average *A. makarovi* female reply (de Groot et al.^22^, Kuhelj et al.^31^)	10.0	430 (0–1970)	Laboratory recording (Kuhelj et al.^31^)
Male advertisement signal	Average *A. makarovi* male advertisement signal (de Groot et al.^22^, Kuhelj et al.^31^)	17.9	172 (55–2710)	Laboratory recording
Masking signal	*A. makarovi* male rival signal, a rapid succession of masking clicks (Kuhelj & Virant-Doberlet^15^)	5.5	344 (0–1990)	Laboratory recording
*A. bicincta* 'Dragonja' signal	»typical« *A. bicincta* 'Dragonja' male advertisement signal (Korinšek et al.^19^)	14.9	94 (0–3120)	Laboratory recording
Anthropogenic noise	Broadband emission of a lawnmower with slowly undulating amplitude (one cycle, approx. 8.5 dB RMS difference between maximum and minimum)	28.0	86 (0–21,000)	Field recording

Signal amplitude was adjusted to the amplitude of male signals emitted from the top of the plant and recorded using a laser vibrometer (PDV 100 or OFV 5000 with OFV 505 sensor head, Polytec GmbH) positioned perpendicularly to the stem and stalks, and directed to the point approximately 5 mm below the crossing where a small piece of reflective foil was glued to the stem. Male signal playback was set to approximately equal amplitude, while all other playbacks were 6 dB quieter to reflect natural conditions^22^.

Stimulation was played through the stereo channels of a computer sound card (various models) controlled with the CoolEdit Pro 2.0 software (Syntrillium Software, 2003). Stimuli and all other vibrations were recorded continuously with a laser vibrometer as described above. Males were also video recorded from the same direction using a video camera (Canon XM2 or Panasonic VXF990). The laser’s output was split into two: one cable was connected with the camera’s microphone jack for synchronization with the video track, while the other was plugged into an external sound card (Sound Blaster XF Surround 5.1 Pro, Creative Technology Ltd., Singapore) connected to a Windows computer. There, it was digitized at 44.1 kHz sample rate using Raven 1.5 software (Cornell Lab of Ornithology, 2017) and stored in an uncompressed Wave format. Headphones plugged in the camera's headphone jack were used for monitoring vibrations in real time.

A male was placed on the top of the cutting and after a few seconds, one male signal-female reply sequence was played to stimulate the male to start singing, except in the “control-” without stimulation. For the “duet” treatment, this sequence was simply looped from the beginning. Signals and their elements were defined according to the existing description^14^, and a female reply playback was triggered manually near the end of each final section (Me3) emitted by the male, when the operator heard that the amplitude started to diminish.

Recording started with the first male signal, and ended at the cutoff time 15 min after that, or when the male located the target point or left the plant, whichever came first. If a male failed to start singing within 5 min, the stimulation was repeated, then again at 10 min. If it remained silent for 15 min, the trial was stopped and repeated at the end of the day, and rejected if the male remained silent the second time. Recording of “duet” treatments started immediately with the first playback which started a few seconds after the male was placed, then the trial continued until the same ending criteria as above were met. Recording of “control–” treatments was the same as “duet”, just without any stimulation.

Each male was tested 5 times during 2 weeks with the same treatment, with 2 or 3 days between consecutive trials and randomized daily order. The *random.org* web service was used for randomization of male order and target designation. Three operators (JP, RJ, AK) were conducting experiments, each responsible for approximately one third of males. Any male that did not survive for the full duration of the experiment was excluded from the analysis, and after 2 weeks, another set of experiments was conducted with newly emerged males from the colony to equalize the size of treatment groups as much as possible. In total, 84 males were analyzed.

#### Trial analysis and statistics

We analyzed the paths of male movements from the video recordings, by manually watching the videos and annotating the movements. To speed up analysis, length of each section of the arena (stem above the crossing, stem below the crossing, stalk, leaf) was divided to ten subsections of equal length, and male’s movement in one direction approximated to the nearest subsection. We measured normal walking separately from searching, the latter could be distinguished by greater speed and having been directly associated with a female reply, i.e. occurring during or immediately after the reply. Movement during negative control trials could thus, by definition, only constitute normal walking. Lateral movement across the leaf was ignored. We also measured latency between the first reply and the onset of moving. Trials in which the male did not start moving after receiving stimulation were excluded from the analysis.

Temporal parameters of signals were analyzed from audio recordings using Raven 1.5. Each signal section was measured separately. A sequence of sections was considered a signal if it included Me3 (during which a reply was triggered) and at least one other section, with less than 2 s silent interval between them; if a signal was interrupted before Me3, the sections were still measured, but not counted as a signal. Similarly, uninterrupted sequences of masking »clicks« were measured as one bout, or individual »clicks« if they occurred alone. All the signal temporal parameters were measured from the spectrogram (Hann filter, FFT window size 4096 samples, 50% overlap) to enable accurate delimitation of faint signals in noisy conditions. Additionally, signaling rhythm and masking of replies were analyzed for the treatment “bionoise” with looped noise playback.

The measured parameters were processed with custom R scripts (built in R version 4.0.2) to calculate derived parameters for assessing overall efficiency (Supplementary Table S2). Those were: duty cycle (total signaling duration / trial duration), path efficiency (starting distance to the target / total traversed distance), and search ratio (distance traversed by searching movements / total traversed distance). Overall efficiency was calculated using the following formula:

$$eff=\frac{1-duty cycle}{\mathrm{log}10\,\, (\frac{distance}{approach})},$$

where “distance” is the total distance traversed by the male and “approach” is the ratio between the male’s closest approach to the target (“ipsi”) point during the whole trial and the male’s starting distance (i.e. remaining at the starting position or moving farther would mean approach = 0 and finding the target point would mean approach = 1). Total signal duration was calculated by adding durations of all marked signal sections in a trial.

We conducted all statistical tests in R version 4.2.2 (R Core Team 2022, Boston, MA, USA), ran in the RStudio (RStudio Team 2022, Boston, MA, USA) interface. To test the prediction that vibrational noise lowers and eavesdropping increases males’ overall efficiency, we fitted linear mixed effects models from the “lme4” package^68^. We fitted full models for overall efficiency, search ratio and path efficiency with treatment as a fixed effect and male ID, plant, repetition and operator as random effects with random intercepts. We chose the best model by stepwise backward elimination strategy and the AIC criterion. We then compared estimated marginal means for treatments based on chosen models with the Tukey method by using the package “emmeans”^69^, or we conducted Tukey HSD after performing ANOVA on linear models^70^. We used the same procedure for the following secondary parameters of interest: total signal duration, number of emitted signals, duty cycle, ratio of rival signal emissions within total signaling, time needed to reach closest position to the target shaker among males who started moving, percent of approach to target, average male signal duration, Me3 ratio, ratio of interrupted signals, moving latency and searching latency. For the proportion of males who found the target shaker, we used a binomial generalized linear mixed-effects model instead.

### Tymbal muscle structure

Two males from “control-” and one each from treatment groups “control + ” and “bionoise” were selected for the analysis of tymbal muscle structure, representing the extremes of low and high signaling effort as determined by the total signaling duration: the selected “control–” males emitted a total 53.8 and 78 s of signal duration in five trials, whereas the “control + ” and “bionoise” males totaled 867.3 and 709.9 s, respectively – a tenfold difference.

The main tymbal muscle (Ia dvm_1_) was examined as follows: the males were anesthetized by low temperature and dissected to remove the abdomen on the same day after the last (fifth) trial. Samples were fixed in 2% formaldehyde and 2.5% glutaraldehyde in 0.1 M Hepes buffer (pH 7.2), rinsed and postfixed in 1% OsO_4_ in the same buffer. After rinsing the samples were dehydrated in ethanol series and acetone, embedded in Agar 100 resin and prepared for sectioning. Semithin sections (0.5 µm) of the first abdominal segment, comprising the longitudinal profiles of the tymbal muscle, were stained with Azure II—methylene blue and imaged by AxioImager Z.1 microscope (Zeiss), equipped with AxioCam HRc camera and AxioVision software. Ultrathin sections were contrasted with uranyl acetate and lead citrate and imaged by CM 100 (Philips) transmission electron microscope, equipped with Orius SC200 camera (Gatan) and Digital Micrograph Suite software. Ultrastructural characteristics were interpreted with respect to each male’s signaling effort during the trials.

### Analysis of oxidative damage

The remaining males used in behavioral trials were dissected on the same day after the last (fifth) trial to obtain tymbal muscle and brain samples. Young males a week after the final moult, never tested, were dissected as control. The samples of each specimen were stored separately in test microtubes at − 80 °C until analysis.

Oxidative damage to proteins in tymbal muscles was assessed by determining carbonyl content using a commercial kit (Protein Carbonyl Content Assay Kit MAK094, Sigma-Aldrich). In this kit, carbonyl content is determined spectrophotometrically by derivatizing protein carbonyl groups with 2,4-dinitrophenylhydrazine (DNPH), resulting in the formation of stable dinitrophenyl (DNP)-hydrazone adducts proportional to the carbonyls present. Homogenates of each tymbal muscle were prepared in 100 µL of 50 mM potassium phosphate buffer (pH 7.4) containing 0.5 mM EDTA and 1 mM PMSF. Homogenates were centrifuged at 13,000 g for 13 min in a refrigerated centrifuge (2K15, Sigma) and the supernatant was used for analysis.

Brain lipid peroxidation was quantified by measuring the concentration of malondialdehyde (MDA). This assay was performed using the Lipid Peroxidation (MDA) Assay Kit (MAK085 Sigma-Aldrich), in which lipid peroxidation is determined by the reaction of MDA with thiobarbituric acid (TBA) to form a product proportional to MDA. The brain of a single animal was used to prepare the homogenate. Protein content was measured using Pierce™ BCA Protein Assay Kit (Thermo Scientific).

All assays were performed according to the manufacturer's recommendations. Absorbance of samples was measured at appropriate wavelengths using a microplate reader (Synergy MX, BioTek). Finally, we computed Pearson’s correlation between indicators of oxidative damage and overall efficiency.

## Supplementary Information

Supplementary Tables.

Supplementary Information 1.

Supplementary Information 2.

Supplementary Legends.

## References

[1] Brumm H, Slabbekoorn H (2005). Acoustic communication in noise. Adv. Study Behav..

[2] Bradbury JW, Vehrencamp SL (2011). Principles of Animal Communication.

[3] Mazzoni V, Eriksson A, Anfora G, Lucchi A, Virant-Doberlet M, Cocroft RB, Gogala M, Hill PSM, Wessel A (2014). Active space and the role of amplitude in plant-borne vibrational communication. Studying Vibrational Communication.

[4] Römer H, Brumm H (2013). Masking by noise in acoustic insects: Problems and solutions. Animal Communication and Noise.

[5] Endler JA, Hill PSM, Lakes-Harlan R, Mazzoni V, Narins PM, Virant-Doberlet M, Wessel A (2019). Biotremology and sensory ecology. Biotremology: Studying Vibrational Behavior.

[6] Virant-Doberlet M, Stritih-Peljhan N, Žunič-Kosi A, Polajnar J (2023). Functional diversity of vibrational signaling systems in insects. Annu. Rev. Entomol..

[7] Michelsen A, Fink F, Gogala M, Traue D (1982). Plants as transmission channels for insect vibrational songs. Behav. Ecol. Sociobiol..

[8] Barth FG, Bleckmann H, Bohnenberger J, Seyfarth E-A (1988). Spiders of the genus Cupiennius Simon 1891 (Araneae, Ctenidae) II. On the vibratory environment of a wandering spider. Oecologia.

[9] Šturm R, Polajnar J, Virant-Doberlet M, Hill PSM, Lakes-Harlan R, Mazzoni V, Narins PM, Virant-Doberlet M, Wessel A (2019). Practical issues in studying natural vibroscape and biotic noise. Biotremology: Studying Vibrational Behavior.

[10] Šturm R, Rexhepi B, López Díez JJ, Blejec A, Polajnar J, Sueur J, Virant-Doberlet M (2021). Hay-meadow vibroscape and interactions within insect vibrational community. iScience.

[11] Polajnar J, Svenšek D, Čokl A (2012). Resonance in herbaceous plant stems as a factor in vibrational communication of pentatomid bugs (Heteroptera: Pentatomidae). J. R. Soc. Interface.

[12] Virant-Doberlet M, Kuhelj A, Polajnar J, Šturm R (2019). Predator-prey interactions and eavesdropping in vibrational communication networks. Front. Ecol. Evol..

[13] Tishechkin DYu (2013). Vibrational background noise in herbaceous plants and its impact on acoustic communication of small *Auchenorrhyncha* and *Psyllinea* (Homoptera). Entomol. Rev..

[14] Derlink M, Pavlovčič P, Stewart AJA, Virant-Doberlet M (2014). Mate recognition in duetting species: The role of male and female vibrational signals. Anim. Behav..

[15] Kuhelj A, Virant-Doberlet M (2017). Male–male interactions and male mating success in the leafhopper *Aphrodes*
*makarovi*. Ethology.

[16] Kuhelj A, De Groot M, Pajk F, Simčič T, Virant-Doberlet M (2015). Energetic cost of vibrational signalling in a leafhopper. Behav. Ecol. Sociobiol..

[17] Claridge MF (1985). Acoustic signals in the Homoptera: Behaviour, taxonomy, and evolution. Annu. Rev. Entomol..

[18] Nickel H, Achtziger R (2005). Do they ever come back? Response of leafhopper communities to extensification of land use. J. Insect Conserv..

[19] Korinšek G, Derlink M, Virant-Doberlet M, Tuma T (2016). An autonomous system of detecting and attracting leafhopper males using species- and sex-specific substrate borne vibrational signals. Comput. Electron. Agric..

[20] Bluemel JK, Derlink M, Pavlovčič P, Russo IM, King RA, Corbett E, Sherrard-Smith E, Blejec A, Wilson MR, Stewart AJA, Symondson WOC, Virant-Doberlet M (2014). Integrating vibrational signals, mitochondrial DNA and morphology for species determination in the genus *Aphrodes* (Hemiptera: Cicadellidae). Syst. Entomol..

[21] Chiykowski LN (1970). Notes on the biology of the leafhopper *Aphrodes*
*bicincta* (Homoptera: Cicadellidae) in the Ottawa area. Can. Entomol..

[22] De Groot M, Derlink M, Pavlovčič P, Prešern J, Čokl A, Virant-Doberlet M (2011). Duetting behaviour in the leafhopper *Aphrodes*
*makarovi* (Hemiptera: Cicadellidae). J. Insect Behav..

[23] Hunt RE, Nault LR (1991). Roles of interplant movement, acoustic communication, and phototaxis in mate-location behavior of the leafhopper *Graminella*
*nigrifrons*. Behav. Ecol. Sociobiol..

[24] Wessel A, Mühlethaler R, Hartung V, Kuštor V, Gogala M, Cocroft RB, Gogala M, Hill PSM, Wessel A (2014). The tymbal: Evolution of a complex vibration-producing organ in the Tymbalia (Hemiptera excl. Sternorrhyncha). Studying Vibrational Communication.

[25] Davranoglou L-R, Mortimer B, Taylor GK, Malenovský I (2020). On the morphology and evolution of cicadomorphan tymbal organs. Arthropod. Struct. Dev..

[26] Nahirney PC, Forbes JG, Morris HD, Chock SC, Wang K (2006). What the buzz was all about: Superfast song muscles rattle the tymbals of male periodical cicadas. FASEB J..

[27] Stokes DR, Josephson RK (2004). Power and control muscles of cicada song: Structural and contractile heterogeneity. J. Comp. Physiol. A.

[28] Syme DA, Josephson RK (2002). How to build fast muscles: Synchronous and asynchronous designs. Integr. Comp. Biol..

[29] De Groot M, Čokl A, Virant-Doberlet M (2010). Effects of heterospecific and conspecific vibrational signal overlap and signal-to noise ratio on male responsiveness in *Nezara*
*viridula* (L.). J. Exp. Biol..

[30] Polajnar J, Eriksson A, Rossi Staconi MV, Lucchi A, Anfora G, Virant-Doberlet M, Mazzoni V (2014). The process of pair formation mediated by substrate-borne vibrations in a small insect. Behav. Proc..

[31] Kuhelj A, De Groot M, Blejec A, Virant-Doberlet M (2016). Sender-receiver dynamics in leafhopper vibrational duetting. Anim. Behav..

[32] Gibson JS, Cocroft RB (2018). Vibration-guided mate searching in treehoppers: Directional accuracy and sampling strategies in a complex sensory environment. J. Exp. Biol..

[33] Eberhard MJB, Metze D, Küpper SC (2019). Causes of variability in male vibratory signals and the role of female choice in Mantophasmatodea. Behav. Process..

[34] McNett GD, Luan LH, Cocroft RB (2010). Wind-induced noise alters signaler and receiver behavior in vibrational communication. Behav. Ecol. Sociobiol..

[35] Sohal RS, Sohal BH, Orr WC (1995). Mitochondrial superoxide and hydrogen peroxide generation, protein oxidative damage, and longevity in different species of flies. Free Radic. Biol. Med..

[36] Archer CR, Sakaluk SK, Selman C, Royle NJ, Hunt J (2013). Oxidative stress and the evolution of sex differences in life span and ageing in the decorated cricket, *Gryllodes*
*sigillatus*. Evolution.

[37] Li-Byarlay H, Huang MH, Simone-Finstrom M, Strand MK, Tarpy DR, Rueppell O (2016). Honey bee (*Apis mellifera*) drones survive oxidative stress due to increased tolerance instead of avoidance or repair of oxidative damage. Exp. Gerontol..

[38] Kramer BH, Nehring V, Buttstedt A, Heinze J, Korb J, Libbrecht R, Meusemann K, Paxton RJ, Séguret A, Schaub F, Bernadou A (2021). Oxidative stress and senescence in social insects: A significant but inconsistent link?. Phil. Trans. R. Soc. B.

[39] Archer CR, Hunt J (2015). Understanding the link between sexual selection, sexual conflict and aging using crickets as a model. Exp. Gerontol..

[40] Simone-Finstrom M, Li-Byarlay H, Huang MH, Strand MK, Rueppell O, Tarpy DR (2016). Migratory management and environmental conditions affect lifespan and oxidative stress in honey bees. Sci. Rep..

[41] Taric E, Glavinic U, Vejnovic B, Stanojkovic A, Aleksic N, Dimitrijevic V, Stanimirovic Z (2020). Oxidative stress, endoparasite prevalence and social immunity in bee colonies kept traditionally vs. those kept for commercial purposes. Insects.

[42] Bulthuis EP, Adjobo-Hermans MJW, Willems PHGM, Koopman WJH (2019). Mitochondrial morphofunction in mammalian cells. Antioxid. Redox Signal..

[43] Jiang Y, Lin S, Chen J, Tsai H, Hsieh T, Fu C (2017). Electron tomographic analysis reveals ultrastructural features of mitochondrial cristae architecture which reflect energetic state and aging. Sci. Rep..

[44] Jayashankar V, Mueller IA, Rafelski SM (2016). Shaping the multi-scale architecture of mitochondria. Curr. Opin. Cell Biol..

[45] Vincent AE, Ng YS, White K, Davey T, Mannella C, Falkous G, Feeney C, Schaefer AM, McFarland R, Gorman GS, Taylor RW, Turnbull DM, Picard M (2016). The spectrum of mitochondrial ultrastructural defects in mitochondrial myopathy. Sci. Rep..

[46] Zick M, Rabl R, Reichert AS (2009). Cristae formation-linking ultrastructure and function of mitochondria. Biochem. Biophys. Acta.

[47] Plecita-Hlavata L, Ježek P (2016). Integration of superoxide formation and cristae morphology for mitochondrial redox signaling. Int. J. Biochem. Cell Biol..

[48] Backdash JZ, Marusich LR (2017). Repeated measures correlation. Front. Psychol..

[49] Eriksson A, Anfora G, Lucchi A, Lanzo F, Virant-Doberlet M, Mazzoni V (2012). Exploitation of insect vibrational signals reveals a new method of pest management. PLoS One.

[50] Polajnar J, Eriksson A, Virant-Doberlet M, Mazzoni V (2016). Mating disruption of a grapevine pest using mechanical vibrations: From laboratory to the field. J. Pest. Sci..

[51] De Groot M, Čokl A, Virant-Doberlet M (2011). Species identity cues: Possibilities for errors during vibrational communication on plant stems. Behav. Biol..

[52] Legendre F, Marting PR, Cocroft RB (2012). Competitive masking of vibrational signals during mate searching in a treehopper. Anim. Behav..

[53] Cocroft RB, Tieu TD, Hoy R, Miles R (2000). Directionality in the mechanical response to substrate vibration in a treehopper (Hemiptera: Membracidae: *Umbonia crassicornis*). J. Comp. Physiol. A.

[54] Prešern J, Polajnar J, De Groot M, Zorović M, Virant-Doberlet M (2018). On the spot: Utilization of directional cues in vibrational communication of a stink bug. Sci. Rep..

[55] Cogliati S, Enriquez JA, Scorrano L (2016). Mitochondrial cristae: Where beauty meets functionality. Trends Biochem. Sci..

[56] Daum B, Walter A, Horst A, Osiewacz HD, Kühlbrandt W (2013). Age-dependent dissociation of ATP synthase dimers and loss of inner-membrane cristae in mitochondria. Proc. Natl. Acad. Sci. U.S.A..

[57] Brandt T, Mourier A, Tain LS, Partridge L, Larsson N-G, Kühlbrandt W (2017). Changes of mitochondrial ultrastructure and function during ageing in mice and *Drosophila*. eLife.

[58] Glancy B, Hartnell LM, Malide D, Yu ZX, Combs CA, Connelly PS, Subramaniam S, Balaban RS (2015). Mitochondrial reticulum for cellular energy distribution in muscle. Nature.

[59] Picard M, McManus MJ, Csordás G, Várnai P, Dorn GW, Williams D, Hajnóczky G, Wallace DC (2015). Trans-mitochondrial coordination of cristae at regulated membrane junctions. Nat. Commun..

[60] Bleck CKE, Kim Y, Willingham TB, Glancy B (2018). Subcellular connectomic analyses of energy networks in striated muscle. Nat. Commun..

[61] Picard M, Gentil BJ, McManus MJ, White K, St Louis K, Gartside SE, Wallace DC, Turnbull DM (2013). Acute exercise remodels mitochondrial membrane interactions in mouse skeletal muscle. J. Appl. Physiol..

[62] Glancy B, Hartnell LM, Combs CA, Femnou A, Sun J, Murphy E, Subramaniam S, Balaban RS (2017). Power grid protection of the muscle mitochondrial reticulum. Cell Rep..

[63] Orci KM, Petróczki K, Barta Z (2016). Instantaneous song modification in response to fluctuating traffic noise in the tree cricket *Oecanthus*
*pellucens*. Anim. Behav..

[64] Gallego-Abenza M, Mathevon N, Wheatcroft D (2020). Experience modulates an insect’s response to anthropogenic noise. Behav. Ecol..

[65] McGregor PK, Catchpole CK, Dabelsteen T, Falls JB, Fusani L, Gerhardt HC, Gilbert F, Horn AG, Klump GM, Kroodsma DE, Lambrechts MM, McComb KE, Nelson DA, Pepperberg IM, Ratcliffe L, Searcy WA, Weary DM, McGregor PK (1992). Design of playback experiments: The Thornbridge Hall NATO ARW consensus. Playback and Studies of Animal Communication.

[66] McGregor PK (2000). Playback experiments: Design and analysis. Acta Ethol..

[67] McNett GD, Miles RN, Homentcovschi D, Cocroft RB (2006). A method for two-dimensional characterization of animal vibrational signals transmitted along plant stems. J. Comp. Physiol. A.

[68] Bates D, Mächler M, Bolker B, Walker S (2015). Fitting linear mixed-effects models using lme4. J. Stat. Softw..

[69] Lenth RV emmeans: Estimated Marginal Means, aka Least-Squares Means. R package version 1.5.1. https://CRAN.R-project.org/package=emmeans (2020).

[70] Yandell BS (1997). Practical Data Analysis for Designed Experiments.

