# Author Correction: Leafhopper males compensate for unclear directional cues in vibration-mediated mate localization

**DOI:** 10.1038/s41598-023-38424-y

**Published:** 2023-07-24

**Authors:** Jernej Polajnar, Anka Kuhelj, Rok Janža, Nada Žnidaršič, Tatjana Simčič, Meta Virant-Doberlet

**Affiliations:** 1 Department of Organisms and Ecosystems Research, National Institute of Biology, Večna pot 111, Ljubljana, Slovenia; 2 Department of Biology, Biotechnical Faculty, University of Ljubljana, Jamnikarjeva 101, Ljubljana, Slovenia

Correction to: *Scientific Reports* 10.1038/s41598-023-35057-z, published online 01 June 2023

The Acknowledgements section in the original version of this Article was incomplete.

“The authors thank Dr. Estefania Velilla for statistical advice, Dr. Rex Cocroft and Dr. Aleš Škorjanc for help with analyzing transmission of vibrations, and Dr. Polona Mrak for help with preparing *Aphrodes* samples for dissection. We also thank students Urša Majerčič and Nastja Fras for assistance with maintaining the laboratory colony of leafhoppers. This study was funded by the Slovenian Research and Innovation Agency (ARIS) through the projects “Vibrational communication networks: from insects to plants” (J1-8142) and “Vibroscape: discovering an overlooked world of vibrational communication” (J1-3016), as well as the core research funding program “Communities, interactions and communications in ecosystems” (P1-0255) awarded to the National Institute of Biology, and the program “Integrative zoology and speleobiology” (P1-0184) awarded to the University of Ljubljana.”

now reads:

“The authors thank Dr. Estefania Velilla for statistical advice, Dr. Rex Cocroft and Dr. Aleš Škorjanc for help with analyzing transmission of vibrations, and Dr. Polona Mrak for help with preparing *Aphrodes* samples for dissection. Dr. Rok Šturm provided the anthropogenic noise sample and assisted with leafhopper collection, handling, and rearing. We also thank students Urša Majerčič and Nastja Fras for assistance with maintaining the laboratory colony of leafhoppers. This study was funded by the Slovenian Research and Innovation Agency (ARIS) through the projects “Vibrational communication networks: from insects to plants” (J1-8142) and “Vibroscape: discovering an overlooked world of vibrational communication” (J1-3016), as well as the core research funding program “Communities, interactions and communications in ecosystems” (P1-0255) awarded to the National Institute of Biology, and the program “Integrative zoology and speleobiology” (P1-0184) awarded to the University of Ljubljana.”

The original Article has been corrected.

