# Supplementary material for: Leafhopper males compensate for unclear directional cues in vibration-mediated mate localization
Source: Sci Rep. 2023 Jun 1;13:8879. doi: 10.1038/s41598-023-35057-z (PMC10235090; doi:10.1038/s41598-023-35057-z)
Supplement: Supplementary file 1 — Supplementary Tables. [file 41598_2023_35057_MOESM1_ESM.docx]

**Leafhopper males compensate for unclear directional cues in vibration-mediated mate localization**

**Jernej Polajnar^*^, Anka Kuhelj, Rok Janža, Nada Žnidaršič, Tatjana Simčič, Meta Virant-Doberlet**

^*^corresponding author; Department of Organisms and Ecosystems Research, National Institute of Biology, Večna pot 111, Ljubljana, Slovenia, e-mail: jernej.polajnar@nib.si

**Supplementary Data S1:** listing and dimensions of nettle shoots used as arenas.

Tab. 1: Lengths of nettle shoot sections used for experiments and transmission tests [mm]. Labels in top row correspond to labels in Fig. 1 in the paper (A: main stem above the stem-leaf crossing, B: left stalk, C: right stalk, D: left leaf, E: right leaf, F: stem below the stem-leaf crossing). JP, RJ and AK denote operators, same as in Suppl. B. Foil: distance between the stem-leaf crossing and the reflective foil on F [mm].

| **Plant** | **A** | **B** | **C** | **D** | **E** | **F** | **foil** | **dates** |
| --- | --- | --- | --- | --- | --- | --- | --- | --- |
| JP1_1 | 106 | 29 | 32 | 79 | 83 | 44 | 7 | 1.-2.7.2019 |
| JP2_1 | 115 | 29 | 32 | 78 | 85 | 44 | 5 | 3.-4.7.2019 |
| JP3_1 | 101 | 35 | 34 | 78 | 81 | 44 | 5 | 5.-6.7.2019 |
| JP4_1 | 109 | 31 | 33 | 81 | 83 | 29 | 5 | 8.-9.7.2019 |
| JP5_1 | 114 | 26 | 28 | 75 | 74 | 43 | 5 | 10.-11.7.2019 |
| JP1_2 | 115 | 31 | 26 | 82 | 72 | 39 | 4 | 15.7.2019 |
| JP2_2 | 99 | 27 | 21 | 73 | 75 | 48 | 6 | 17.7.2019 |
| JP3_2 | 111 | 19 | 30 | 70 | 72 | 48 | 5 | 19.7.2019 |
| JP4_2 | 97 | 20 | 26 | 68 | 72 | 41 | 6 | 22.7.2019 |
| JP5_2 | 116 | 24 | 24 | 73 | 82 | 44 | 6 | 24.7.2019 |
| RJ1 | 134 | 25 | 22 | 90 | 88 | 45 |  | 8.-9.7.2019 |
| RJ2 | 114 | 33 | 33 | 108 | 116 | 30 |  | 10.-11.7.2019 |
| RJ3 | 117 | 52 | 40 | 115 | 113 | 33 |  | 12.7.-13.2019 |
| RJ4 | 118 | 33 | 35 | 110 | 110 | 30 |  | 15.-16.7.2019 |
| RJ5 | 124 | 32 | 34 | 108 | 112 | 32 |  | 17.-18.7.2019 |
| AK1 | 130 | 24 | 25 | 81 | 83 | 45 | 5 | 8.-9.7.2019 |
| AK2 | 125 | 30 | 31 | 94 | 91 | 47 | 3 | 10.-11.7.2019 |
| AK3 | 121 | 33 | 34 | 93 | 89 | 45 | 7 | 12.7.-13.2019 |
| AK4 | 132 | 35 | 30 | 107 | 106 | 45 | 7 | 15.-16.7.2019 |
| AK5 | 124 | 36 | 34 | 95 | 93 | 44 | 6 | 17.-18.7.2019 |
| transm_test 1 | 143 | 37 | 41 | 69 | 76 | 38 | 3 |  |
| transm_test 2 | 115 | 41 | 44 | 62 | 68 | 36 | 5 |  |
| transm_test 3 | 97 | 27 | 27 | 68 | 74 | 34 | 3 |  |
| transm_test 4 | 96 | 44 | 47 | 57 | 67 | 25 | 3 |  |

Tab. 2: IDs of males tested on each nettle shoot (male and repetition labels correspond to those in Suppl. B).

| **Plant** | **repetition** | **males** | **dates** |
| --- | --- | --- | --- |
| JP1_1 | 1 | a_6, a_7, a_8, a_9, b_6, b_7, b_8, b_9, c_5, c_6, c_7, c_8, c_9, d_5, d_6, d_7, d_9, e_6, e_7, e_8, e_9, e_10, k_6, k_7, k_8, k_9, k_10 | 1.-2.7.2019 |
| JP2_1 | 2 |  | 3.-4.7.2019 |
| JP3_1 | 3 |  | 5.-6.7.2019 |
| JP4_1 | 4 |  | 8.-9.7.2019 |
| JP5_1 | 5 |  | 10.-11.7.2019 |
| JP1_2 | 1 | a_17, a_18, a_19, a_20, d_15 | 15.7.2019 |
| JP2_2 | 2 |  | 17.7.2019 |
| JP3_2 | 3 |  | 19.7.2019 |
| JP4_2 | 4 |  | 22.7.2019 |
| JP5_2 | 5 |  | 24.7.2019 |
| RJ1 | 1 | a_1, a_2, a_3, a_4, a_5, b_1, b_2, b_3, b_4, b_5, c_1, c_2, c_3, c_4, d_1, d_2, d_3, d_4, e_1, e_2, e_3, e_4, e_5, k_1, k_2, k_3, k_4, k_5 | 8.-9.7.2019 |
| RJ2 | 2 |  | 10.-11.7.2019 |
| RJ3 | 3 |  | 12.7.-13.2019 |
| RJ4 | 4 |  | 15.-16.7.2019 |
| RJ5 | 5 |  | 17.-18.7.2019 |
| AK1 | 1 | a_11, a_12, a_15, a_16, b_10, b_11, b_12, b_13, b_14, b_15, c_10, c_11, c_12, c_13, c_14, c_15, d_10, d_11, d_12, d_13, d_14, e_11, e_12, e_13, e_14, e_15, k_11, k_12, k_13, k_14, k_16 | 8.-9.7.2019 |
| AK2 | 2 |  | 10.-11.7.2019 |
| AK3 | 3 |  | 12.7.-13.2019 |
| AK4 | 4 |  | 15.-16.7.2019 |
| AK5 | 5 |  | 17.-18.7.2019 |

Tab. 3: amplitude of the major axis of vibration of nettle cuttings excited through the tip of the left (ipsilateral) leaf, relative to the value at below the steam/stalk crossing (F1). See Fig. 1 for diagram of locations and Online Resource 1 for lengths of sections.

| **Section** | **Location** | **Amplitude [dB]** | | | | |
| --- | --- | --- | --- | --- | --- | --- |
|  |  | **Nettle 1** | **Nettle 2** | **Nettle 3** | **Nettle 4** | **Average** |
| Stem - top | A1 | 0.27 | 0.23 | -2.52 | 2.55 | 0.99 |
|  | A2 | 2.65 | -6.78 | -5.16 | 6.71 | 2.05 |
|  | A3 | -3.89 | -0.04 | 0.19 | 2.48 | 0.63 |
|  | A4 | -2.16 | -7.62 | -2.76 | 7.66 | 2.15 |
|  | A5 | 5.46 | -5.73 | -4.03 | 6.58 | 2.68 |
| Stalk – ipsi | B1 | -11.90 | 4.24 | 8.50 | 7.86 | 5.37 |
|  | B2 | - | 16.26 | 18.35 | 12.53 | 16.16 |
|  | B3 | 6.61 | 3.93 | 18.43 | 13.95 | 11.32 |
| Stalk - contra | C1 | 3.90 | -1.64 | -2.22 | -0.41 | -0.15 |
|  | C2 | 5.61 | 4.39 | -4.09 | -0.43 | 2.37 |
|  | C3 | 6.06 | 1.78 | -19.41 | 8.97 | 5.32 |
| Stem - bottom | F1 | 0.00 | 0.00 | 0.00 | 0.00 | 0.00 |
|  | F2 | -4.89 | -0.54 | -6.58 | -1.93 | -2.14 |
| Leaves | L1-ipsi | 2.35 | 12.17 | 30.94 | 15.41 | 17.59 |
|  | L2- ipsi | 6.38 | 13.60 | 25.77 | -3.66 | 12.64 |
|  | L1-contra | -6.09 | -5.18 | -6.37 | -3.66 | -4.79 |
|  | L2-contra | -9.40 | 3.36 | 0.66 | 0.97 | 1.00 |
