# Supplementary material for: Leafhopper males compensate for unclear directional cues in vibration-mediated mate localization
Source: Sci Rep. 2023 Jun 1;13:8879. doi: 10.1038/s41598-023-35057-z (PMC10235090; doi:10.1038/s41598-023-35057-z)

**Leafhopper males compensate for unclear directional cues in vibration-mediated mate localization**

**Jernej Polajnar^*^, Anka Kuhelj, Rok Janža, Nada Žnidaršič, Tatjana Simčič, Meta Virant-Doberlet**

^*^corresponding author; Department of Organisms and Ecosystems Research, National Institute of Biology, Večna pot 111, Ljubljana, Slovenia, e-mail: jernej.polajnar@nib.si

**Supplementary Data S3:** results of post-hoc tests for secondary parameters.

**Table 1:** the estimated marginal means evaluated in the Tukey test based on linear mixed-effect model for overall efficiency including treatment as a fixed effect and random intercepts for different male.ID and plant. Bolded values denote significant mean differences at α = 0.05.

| contrast | estimate | SE | df | t.ratio | p.value |
| --- | --- | --- | --- | --- | --- |
| **»control+« – »rival«** | -0.019 | 0.006 | 71.0 | -3.494 | **0.0071** |
| **»control+« – »duet«** | -0.057 | 0.006 | 74.5 | -10.237 | **<.0001** |
| **»control+« – »bionoise«** | -0.031 | 0.006 | 73.2 | -5.389 | **<.0001** |
| »control+« – »anthronoise« | -0.011 | 0.006 | 73.5 | -1.986 | 0.2832 |
| **»rival« – »duet«** | -0.038 | 0.005 | 69.7 | -7.105 | **<.0001** |
| »rival« – »bionoise« | -0.012 | 0.006 | 69.1 | -2.148 | 0.2121 |
| »rival« – »anthronoise« | 0.008 | 0.005 | 69.0 | 1.531 | 0.5464 |
| »duet« – »bionoise« | 0.026 | 0.006 | 72.2 | 4.616 | 0.0002 |
| **»duet« – »anthronoise«** | 0.046 | 0.005 | 72.4 | 8.528 | **<.0001** |
| **»bionoise« – »anthronoise«** | 0.020 | 0.006 | 71.8 | 3.568 | **0.0056** |

**Table 2:** the estimated marginal means evaluated in the Tukey test based on linear mixed-effect model for search/walking ratio among males who started moving including treatment as a fixed effect and random intercepts for for different male.ID and plant. Bolded values denote significant mean differences at α = 0.05.

| contrast | estimate | SE | df | t.ratio | p.value |
| --- | --- | --- | --- | --- | --- |
| »control+« – »rival« | 0.004 | 0.057 | 62.1 | 0.070 | 1 |
| »control+« – »duet« | 0.055 | 0.059 | 65.4 | 0.933 | 0.8829 |
| »control+« – »bionoise« | 0.031 | 0.060 | 64.3 | 0.505 | 0.9866 |
| **»control+« – »anthronoise«** | 0.239 | 0.058 | 65.0 | 4.125 | **0.0010** |
| »rival« – »duet« | 0.051 | 0.056 | 60.3 | 0.903 | 0.8946 |
| »rival« – »bionoise« | 0.027 | 0.058 | 59.8 | 0.454 | 0.9910 |
| **»rival« – »anthronoise«** | 0.236 | 0.056 | 60.2 | 4.233 | **0.0007** |
| »duet« – »bionoise« | -0.024 | 0.060 | 62.7 | -0.410 | 0.9939 |
| **»duet« – »anthronoise«** | 0.184 | 0.060 | 63.4 | 3.232 | **0.0162** |
| **»bionoise« – »anthronoise«** | 0.208 | 0.060 | 62.7 | 3.540 | **0.0066** |

**Table 3:** the estimated marginal means evaluated in the Tukey test based on linear mixed-effect model for number of emitted male calls including treatment as a fixed effect and random intercepts for different male.ID. Bolded values denote significant mean differences at α = 0.05.

| contrast | estimate | SE | df | t.ratio | p.value |
| --- | --- | --- | --- | --- | --- |
| »control+« – »rival« | 2.994 | 2.30 | 66.1 | 1.299 | 0.6928 |
| **»control+« – »duet«** | 22.313 | 2.34 | 65.3 | 9.549 | **<.0001** |
| **»control+« – »bionoise«** | 7.810 | 2.39 | 66.7 | 3.262 | **0.0146** |
| »control+« – »anthronoise« | -0.222 | 2.30 | 66.1 | -0.097 | 1.0000 |
| **»rival« – »duet«** | 19.320 | 2.30 | 65.2 | 8.412 | **<.0001** |
| »rival« – »bionoise« | 4.817 | 2.36 | 66.6 | 2.045 | 0.2565 |
| »rival« – »anthronoise« | -3.216 | 2.26 | 66.0 | -1.420 | 0.6169 |
| **»duet« – »bionoise«** | -14.503 | 2.39 | 65.9 | -6.076 | **<.0001** |
| **»duet« – »anthronoise«** | -22.536 | 2.30 | 65.2 | -9.812 | **<.0001** |
| **»bionoise« – »anthronoise«** | -8.033 | 2.36 | 66.6 | -3.410 | **0.0095** |

**Table 4:** the estimated marginal means evaluated in the Tukey test based on linear mixed-effect model for total signal duration including treatment as a fixed effect and random intercepts for different male.ID. Bolded values denote significant mean differences at α = 0.05.

| contrast | estimate | SE | df | t.ratio | p.value |
| --- | --- | --- | --- | --- | --- |
| »control+« – »rival« | 56.1 | 27.5 | 66.1 | 2.040 | 0.2586 |
| **»control+« – »duet«** | 246.4 | 27.9 | 65.3 | 8.840 | **<.0001** |
| **»control+« – »bionoise«** | 100.0 | 28.6 | 66.6 | 3.501 | **0.0072** |
| »control+« – »anthronoise« | 14.9 | 27.5 | 66.1 | 0.542 | 0.9826 |
| **»rival« – »duet«** | 190.3 | 27.4 | 65.2 | 6.947 | **<.0001** |
| »rival« – »bionoise« | 43.9 | 28.1 | 66.6 | 1.563 | 0.5261 |
| »rival« – »anthronoise« | -41.2 | 27.0 | 66.0 | -1.526 | 0.5497 |
| **»duet« – »bionoise«** | -146.4 | 28.5 | 65.9 | -5.143 | **<.0001** |
| **»duet« – »anthronoise«** | -231.5 | 27.4 | 65.2 | -8.451 | **<.0001** |
| **»bionoise« – »anthronoise«** | -85.1 | 28.1 | 66.6 | -3.030 | **0.0278** |

**Table 5:** the estimated marginal means evaluated in the Tukey test based on linear mixed-effect model for duty cycle including treatment as a fixed effect and random intercepts for different male.ID and plant. Bolded values denote significant mean differences at α = 0.05.

| contrast | estimate | SE | df | t.ratio | p.value |
| --- | --- | --- | --- | --- | --- |
| »control+« – »rival« | 0.082 | 0.033 | 66.3 | 2.638 | 0.0750 |
| **»control+« – »duet«** | 0.351 | 0.034 | 65.9 | 10.453 | **<.0001** |
| **»control+« – »bionoise«** | 0.157 | 0.034 | 65.5 | 4.586 | **0.0002** |
| »control+« – »anthronoise« | 0.071 | 0.033 | 66.2 | 2.138 | 0.2167 |
| **»rival« – »duet«** | 0.264 | 0.033 | 64.1 | 8.062 | **<.0001** |
| »rival« – »bionoise« | 0.069 | 0.034 | 65.0 | 2.069 | 0.2461 |
| »rival« – »anthronoise« | -0.017 | 0.032 | 64.4 | -0.515 | 0.9856 |
| **»duet« – »bionoise«** | -0.195 | 0.034 | 64.5 | -5.729 | **<.0001** |
| **»duet« – »anthronoise«** | -0.281 | 0.033 | 63.9 | -8.581 | **<.0001** |
| »bionoise« – »anthronoise« | -0.086 | 0.034 | 64.9 | -2.565 | 0.0891 |

**Table 6:** the estimated marginal means evaluated in the Tukey test based on linear mixed-effect model for proportion of masking signal duration within total signaling duration, including random intercepts for different male.ID. Bolded values denote significant mean differences at α = 0.05.

| contrast | estimate | SE | df | t.ratio | p.value |
| --- | --- | --- | --- | --- | --- |
| »control+« – »rival« | 0.017 | 0.061 | 63.2 | 0.287 | 0.9985 |
| **»control+« – »duet«** | -0.432 | 0.065 | 68.3 | -6.642 | **<.0001** |
| »control+« – »bionoise« | -0.004 | 0.064 | 63.4 | -0.055 | 1 |
| »control+« – »anthronoise« | -0.006 | 0.061 | 63.2 | -0.099 | 1 |
| **»rival« – »duet«** | -0.450 | 0.064 | 68.4 | -7.023 | **<.0001** |
| »rival« – »bionoise« | -0.021 | 0.063 | 63.4 | -0.338 | 0.9971 |
| »rival« – »anthronoise« | -0.024 | 0.060 | 63.2 | -0.393 | 0.9948 |
| **»duet« – »bionoise«** | 0.428 | 0.066 | 68.2 | 6.471 | **<.0001** |
| **»duet« – »anthronoise«** | 0.426 | 0.064 | 68.4 | 6.652 | **<.0001** |
| »bionoise« – »anthronoise« | -0.003 | 0.063 | 63.4 | -0.041 | 1 |

**Table 7:** Tukey test based on marginal means after ANOVA on linear model for the time needed to reach closest position to the target shaker among males who started moving, including treatment as predictor. Bolded values denote significant mean differences at α = 0.05.

| contrast | diff | lwr | upr | p.value |
| --- | --- | --- | --- | --- |
| »rival« – »control+« | 24.008 | -81.328 | 129.343 | 0.971 |
| »duet« – »control+« | -57.584 | -167.302 | 52.134 | 0.602 |
| »bionoise« – »control+« | 73.101 | -39.374 | 185.577 | 0.385 |
| »anthronoise« – »control+« | 32.632 | -76.102 | 141.366 | 0.923 |
| »duet« – »rival« | -81.592 | -187.942 | 24.759 | 0.220 |
| »bionoise« – »rival« | 49.094 | -60.099 | 158.287 | 0.731 |
| »anthronoise« – »rival« | 8.624 | -96.711 | 113.959 | 0.999 |
| **»bionoise« – »duet«** | 130.686 | 17.259 | 244.112 | **0.015** |
| »anthronoise« – »duet« | 90.216 | -19.502 | 199.934 | 0.162 |
| »anthronoise« – »bionoise« | -40.470 | -152.945 | 72.005 | 0.861 |

**Table 8:** Tukey test based on marginal means after ANOVA on linear model for the proportion of interrupted calls, including treatment as predictor. Bolded values denote significant mean differences at α = 0.05.

| contrast | diff | lwr | upr | p.value |
| --- | --- | --- | --- | --- |
| »rival« – »control+« | -0.0134 | -0.072 | 0.045 | 0.970 |
| **»duet« – »control+«** | 0.1919 | 0.111 | 0.273 | **<.0001** |
| »bionoise« – »control+« | 0.0177 | -0.043 | 0.079 | 0.932 |
| »anthronoise« – »control+« | -0.0196 | -0.078 | 0.039 | 0.888 |
| **»duet« – »rival«** | 0.2053 | 0.125 | 0.286 | **<.0001** |
| »bionoise« – »rival« | 0.0311 | -0.029 | 0.091 | 0.612 |
| »anthronoise« – »rival« | -0.0062 | -0.064 | 0.051 | 0.998 |
| **»bionoise« – »duet«** | -0.1742 | -0.256 | -0.092 | **<.0001** |
| **»anthronoise« – »duet«** | -0.2115 | -0.292 | -0.131 | **<.0001** |
| »anthronoise« – »bionoise« | -0.0373 | -0.097 | 0.023 | 0.430 |

**Figure 1:** graphs of models for parameters in Tabs. 3-8. Estimated marginal means in black and arithmetic means in blue, where applicable.


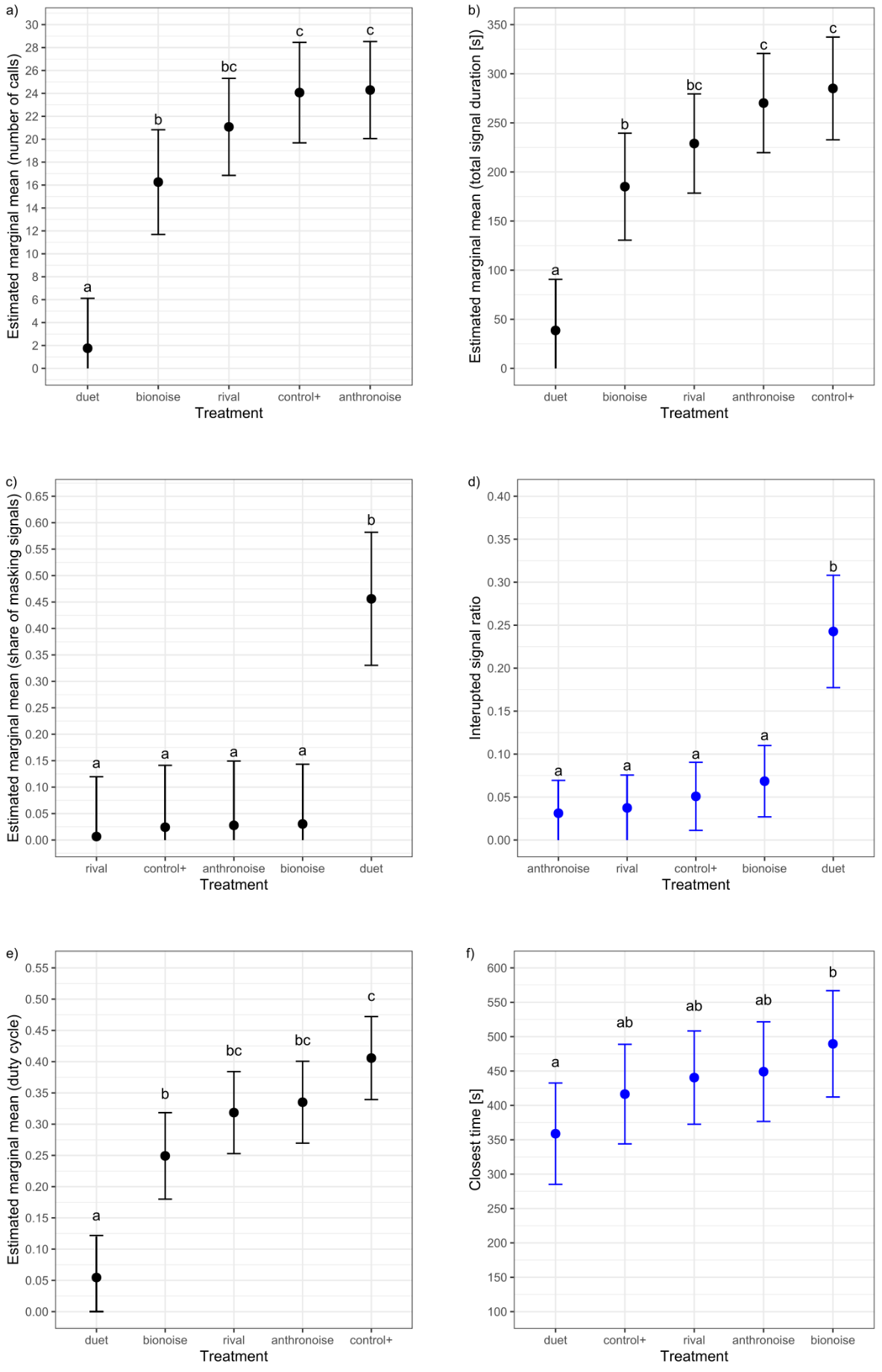


**Figure 2:** box-and-whiskers plots or dot-plots for parameters where treatment was not found to have an effect (red: median, blue: mean).


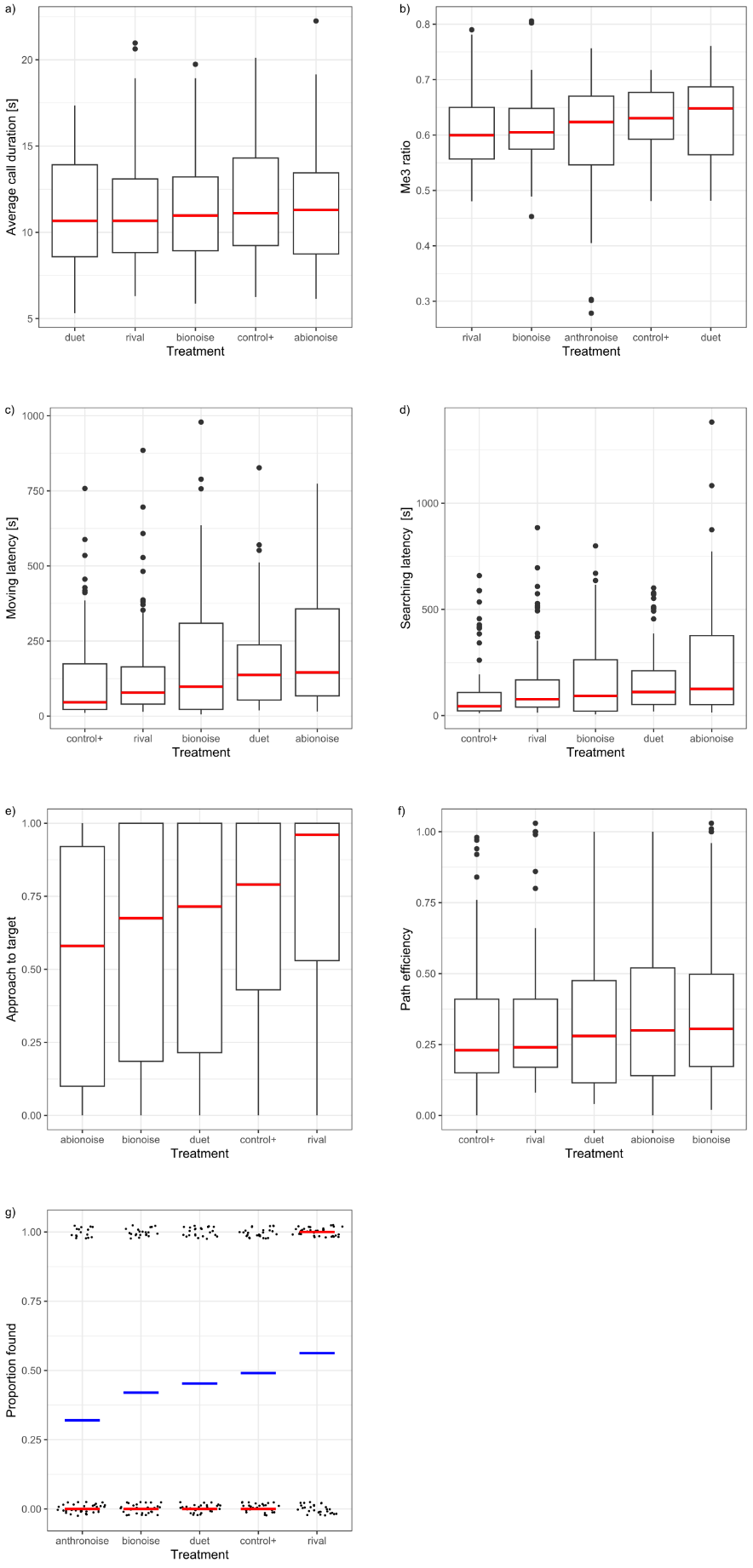

Supplement: Supplementary file 3 — Supplementary Information 2. [file 41598_2023_35057_MOESM3_ESM.docx]
