# Supplementary material for: Leafhopper males compensate for unclear directional cues in vibration-mediated mate localization
Source: Sci Rep. 2023 Jun 1;13:8879. doi: 10.1038/s41598-023-35057-z (PMC10235090; doi:10.1038/s41598-023-35057-z)
Supplement: Supplementary file 4 — Supplementary Legends. [file 41598_2023_35057_MOESM4_ESM.docx]

Supplementary data S2: partially processed raw data from behavioral trials, with secondary parameters calculated. In column labels, the suffix “_search” refers to searching movements only, and “_all” to all movements (searching included). Labels denote: “start.t” and “start.distance” = time and distance from the target when the first signal was recorded; “latency” = time from first signal to start of moving; “approach”: starting/closest distance to target achieved; “closest.t” = time when closest distance to target was achieved; “trial_duration” = time between start of signaling to end of trial; “distance” = total distance traversed by the male; “path.eff” = distance/start.distance; “turns” = number of direction reversals; “started” and “found” = did the male start moving and find the target?; “signalduration” = sum of all durations of signal emissions during the trial; “searchratio” = distance_search/distance_all; “dutycycle” = signalduration/trial_duration; “rivdutycycle” = sum of durations of masking signals/trial_duration; “no.signals” = number of complete signals; “signalduration.avg” = signalduration/no.signals; “rivratio.tot” = sum of durations of masking signals/signalduration; “no.interrupted.signals” = number of signals without Me3 (not counted in “no.signals”); “interrupted.signals.ratio” = no.interrupted.signals/(no.signals+no.interrupted.signals); “XXratio.sig” = sum of durations of each signal section/signalduration; “eff” = overall efficiency.
